# Expression and prognostic significance of CCL11/CCR3 in glioblastoma

**DOI:** 10.18632/oncotarget.8958

**Published:** 2016-04-23

**Authors:** Min Tian, Lina Chen, Li Ma, Dandan Wang, Bin Shao, Jianyu Wu, Hangyu Wu, Yimin Jin

**Affiliations:** ^1^ Department of Gerontology, First Affiliated Hospital of Harbin Medical University, Harbin 150001, China; ^2^ Department of General Surgery, First Affiliated Hospital of Harbin Medical University, Harbin 150001, China; ^3^ Department of Emergency, the General Hospital of Beijing Military Command, Beijing 100700, China

**Keywords:** glioblastoma, CCL11, CCR3, prognosis, biomarker

## Abstract

Glioblastoma (GBM) is the most lethal primary nervous system cancer, but due to its rarity and complexity, its pathogenesis is poorly understood. To identify potential tumorigenic factors in GBM, we screened antibody-based cytokine arrays and found that CCL11 was upregulated. We then demonstrated *in vitro* that both CCL11 and its receptor, CCR3, were overexpressed and promoted the proliferation, migration and invasion of cancer cells. To examine the clinical significance of CCL11/CCR3, 458 GBM samples were divided into a training cohort with 225 cases and a test cohort containing 233 cases. In the training set, immunohistochemical analysis showed overexpression of CCL11 and CCR3 were correlated with unfavorable overall survival (OS). We further developed a prognostic classifier combining CCL11 and CCR3 expression and Karnofsky performance status (KPS) for predicting one-year survival in GBM patients. Receiver operating characteristic (ROC) analysis demonstrated that this predictor achieved 90.7% sensitivity and 73.4% specificity. These results were validated with the test sample set. Our findings suggest that CCL11-CCR3 binding is involved in the progression of GBM and may prompt a novel therapeutic approach. In addition, CCL11 and CCR3 expression, combined with KPS, may be used as an accurate predictor of one-year survival in GBM patients.

## INTRODUCTION

Glioblastoma multiforme (GBM) is the most fatal primary brain tumor. Due to the poor efficacy of conventional therapeutics, the median survival period of GBM patients is only about one year [1]. Chemokines are a family of secreted proteins that act though autocrine or paracrine fashion, and they are thought to influence tumor development [2]. CCL11, also known as eotaxin-1, is an eosinophil-selective chemoattractant cytokine. It is widely expressed in human tissues including heart, colon, kidney, small intestine, lung, pancreas, liver, and ovary [3, 4]. CCL11 primarily binds to CCR3, a seven-transmembrane-domain G-protein-coupled chemokine receptor [5]. This binding triggers a series of signal transduction events involving transient release of intracellular calcium, cytoskeletal rearrangements, generation of inositol triphosphate, activation of protein kinase C and prolonged receptor internalization into an endocytic compartment [5–7]. In cancer, CCL11 and CCR3 facilitate proliferation, migration, and invasion of cancer cells [5, 8–13]. Indirect evidence also suggests that CCL11 may participate in angiogenesis and metastasis [14]. However, the association between CCL11/CCR3 and GBM has not been adequately investigated.

In this study, we first analyzed the cytokine profiles of clinical GBM tissues and discovered that CCL11 was a potential GBM tumor biomarker. We then examined the expression of CCL11 in several cell lines and human GBM samples, and investigated whether CCL11 and CCR3 contributed to the proliferation, migration and invasion of cancer cells *in vitro*. We further analyzed the correlation between CCL11/CCR3 expression with other clinic-pathologic characteristics, and developed a prognostic classifier combining expression of CCL11 and CCR3 with Karnofsky performance status (KPS) for predicting one-year survival in GBM patients. Our primary goals were to explore the clinical significance of CCL11/CCR3 in tumor progression, and to find out valuable prognostic and predictive biomarkers in GBM.

## RESULTS

### Overexpression of CCL11 and CCR3 in GBM tissues and cell lines

**Figure 1:**
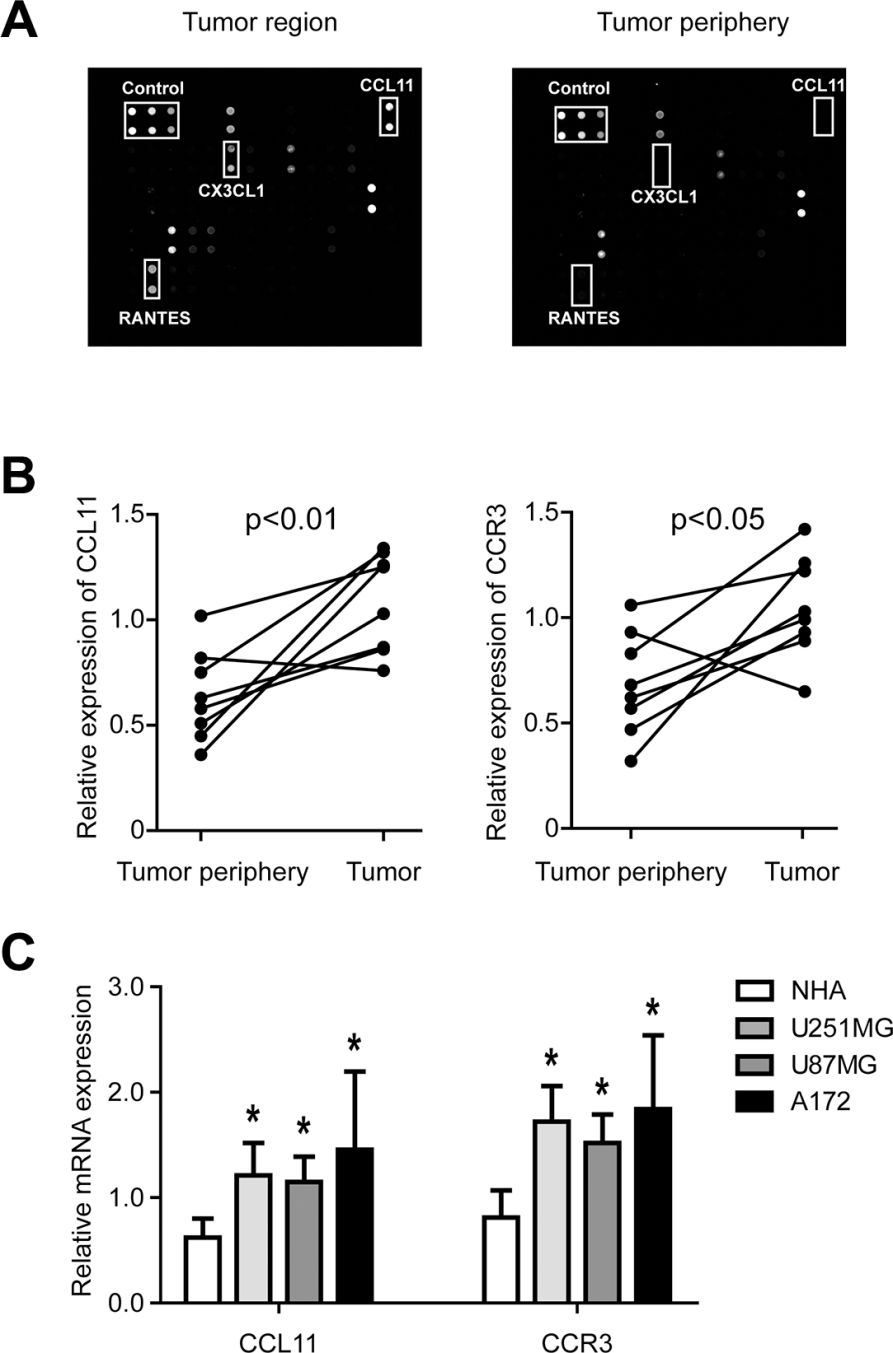
CCL11 and CCR3 were upregulated in GBM (**A**) Antibody-based cytokine array analysis from clinical specimens demonstrated CCL11 levels were increased in tumors compared with adjacent tissues. (**B**) CCL11 and CCR3 mRNA expression in tumors and their adjacent tissues as measured by qRT-PCR (*n* = 8). (**C**) CCL11 and CCR3 mRNA expression in three GBM cell lines (U251MG, U87MG and A172) were upregulated compared with control cells (normal human astrocytes; NHA).*p < 0.05.

To identify potential tumorigenic factors in GBM, the expression profiles of 60 cytokines from clinical samples with their adjacent tissues were examined by an antibody-based cytokine array system. As shown in Figure 1A, the levels of several cytokines including CX3CL1, RANTES, and CCL11 were upregulated in GBM tumors. Since previous studies had shown that RANTES [15] and CX3CL1 [16] expression were correlated with the development of GBM, we focused on elucidating the role of CCL11. Real-time quantitative PCR demonstrated that CCL11 was overexpressed in 8 freshly collected GBM samples compared with paired adjacent tissue from the same subject (Figure 1B, left panel). In addition, expression of CCR3, the receptor for CCL11, was also increased in the tumor (Figure 1B, right panel). Expression of CCL11 and CCR3 was also examined in normal human astrocytes (NHA) and three different glioma cell lines (U251MG, U87MG and A172). Compared with NHA, the levels of both CCL11 and CCR3 were increased in cancer cells (Figure 1C).

### CCL11/CCR3 promote tumor cell proliferation, migration, and invasion

**Figure 2:**
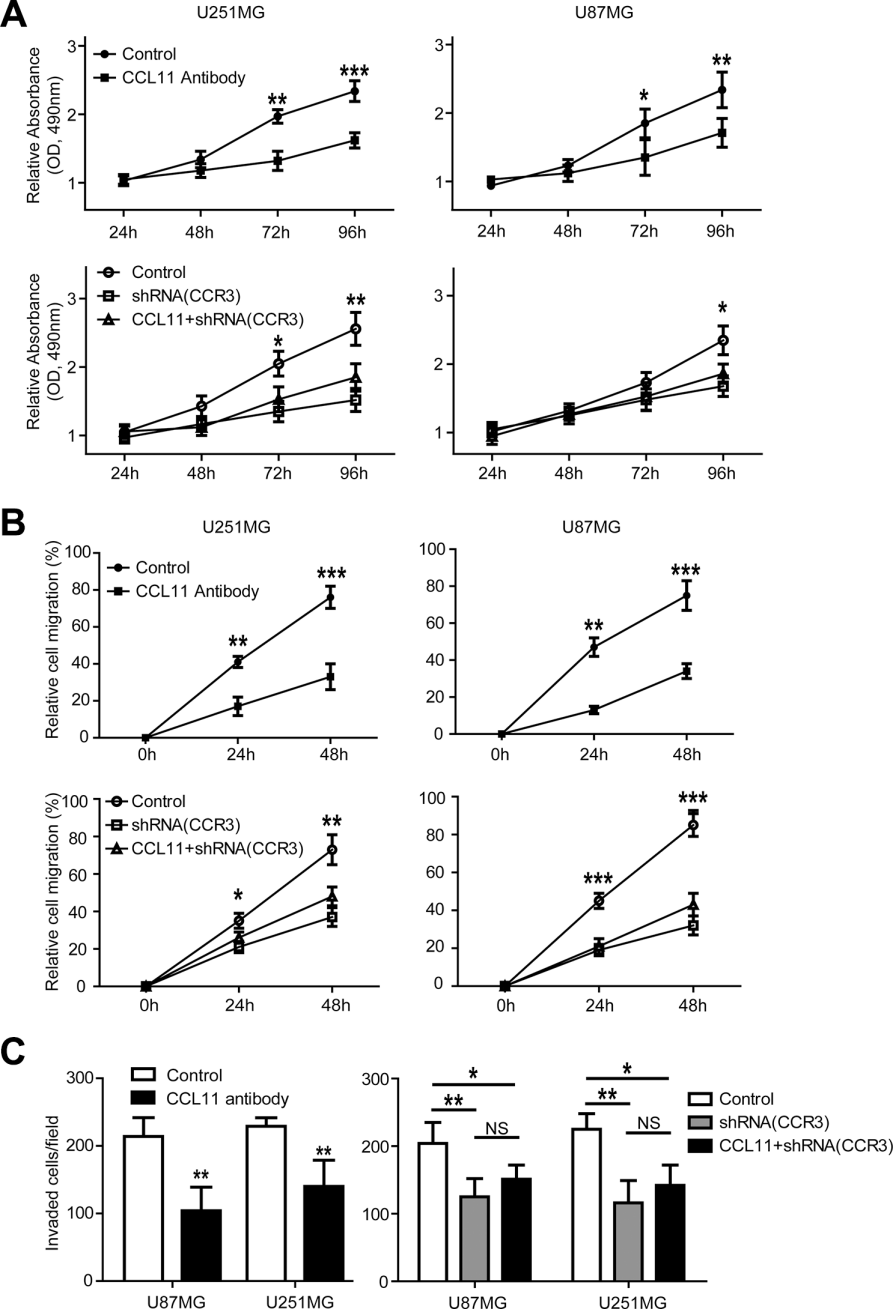
CCL11/CCR3 stimulated proliferation, migration, and invasion in GBM cells (**A**) MTT assay at various time-points revealed that both CCL11 antibody and *CCR3*-shRNA inhibit the proliferation of cancer cells. The inhibitory effect of *CCR3*-shRNA could not be reversed by adding CCL11. (**B**) Wound healing assay at different time-points demonstrated that both CCL11 antibody and *CCR3*-shRNA inhibited the migration of cancer cells. The inhibitory effect of *CCR3*-shRNA could not be reversed by adding CCL11. (**C**) Invasion assay illustrated that both CCL11 antibody and *CCR3*-shRNA inhibited cancer cell invasion. The inhibitory effect of *CCR3*-shRNA could not be reversed by adding CCL11. *p < 0.05; **p < 0.01; ***p < 0.001.

To explore the effects of CCL11 and CCR3 on cell proliferation, cell lines were cultured with CCL11 antibody and cell viability measured by MTT assays. As shown in Figure 2A, CCL11 antibody inhibited cell growth by 31% in U251MG cells and 27% in U87MG cells after 96 hours (Figure 2A, upper panel). Similarly, silencing the *CCR3* gene with shRNA weakened cell proliferation by 39% in U251MG cells and 28% in U87MG cells. Moreover, cell growth could not be restored with the addition of CCL11 (Figure 2A, lower panel).

To address the impact of CCL11 and CCR3 on the motility of cancer cells, wound-healing assays were conducted. As illustrated in Figure 2B, cells cultured with CCL11 antibody were slower to migrate as compared with controls. Quantification of wound closure showed that after 48 hours, cells cultured with CCL11 antibody closed 56% of the wound in U87MG cells and 57% of the wound in U251MG cells (Figure 2B, upper panel). As expected, silencing *CCR3* inhibited the migration of cultured cells and migration could not be restored with CCL11 (Figure 2B, lower panel).

Finally, the importance of CCL11/CCR3 for cell invasiveness was examined with transwell invasion assays. CCL11 antibody inhibited cell invasion by 51% in U87MG cells and 39% in U251MG cells after 48 hours (Figure 2C, left panel). Silencing *CCR3* also weakened the invasion ability of cancer cells, and it could not be restored by CCL11 add back (Figure 2C, right panel).

### Overexpression of CCL11 and CCR3 correlates with poor overall survival

To investigate the clinical role of CCL11 and CCR3 in GBM, their expression was evaluated in patient samples by immunohistochemical staining. A total of 458 GBM patients were enrolled, the median age was 47.3 years (range, 16–85). 255 (56%) subjects were males and 203 (44%) were females. Median follow-up was 12.8 months. The clinical and pathologic characteristics of the patient population are described in Table 1.

**Table 1:** Association of CCL11 expression with clinicopathological characteristics in GBM patients

Variable	Training set (*n* = 225)			Testing set (*n* = 233)		
	High expression	Low expression	*p*	High expression	Low expression	*p*
**Age, years**			0.70			0.59
<= 60	55 (24.4%)	80 (35.6%)		60 (25.8%)	83 (35.6%)	
> 60	39 (17.3%)	51 (22.7%)		41 (17.6%)	49 (21.0%)	
**Gender**			0.77			0.89
Male	52 (23.1%)	75 (33.3%)		56 (24.0%)	72 (30.9%)	
Female	42 (18.7%)	56 (24.9%)		45 (19.3%)	60 (25.8%)	
**Family history of cancer**			0.86			0.88
yes	21 (9.3%)	28 (12.4%)		24 (10.3%)	30 (12.9%)	
no	73 (32.4%)	103 (45.8%)		77 (33.0%)	92 (39.5%)	
**Previous low-grade tumor**			0.41			0.26
yes	4 (1.8%)	9 (4.0%)		4 (1.7%)	10 (4.3%)	
no	90 (40.0%)	122 (54.2%)		97 (41.6%)	122 (52.4%)	
**KPS**			**0.03**			**0.04**
<= 70	63 (28.0%)	69 (30.7%)		65 (27.9%)	67 (28.8%)	
> 70	31 (13.8%)	62 (27.6%)		36 (15.5%)	65 (27.9%)	
**Extent of surgery**			0.64			0.48
Complete resection	56 (24.9%)	79 (35.1%)		62 (26.6%)	87 (37.3%)	
Partial resection	38 (16.9%)	52 (23.1%)		39 (16.7%)	45 (19.3%)	
**Diameter of tumor**			0.15			0.10
<= 5 cm	39 (17.3%)	67 (29.8%)		37 (15.9%)	62 (26.6%)	
> 5 cm	55 (24.4%)	64 (28.4%)		64 (27.5%)	70 (30.0%)	
**CCR3 immunostaining**			**0.0001**			**0.0001**
High expression	69 (30.7%)	41 (18.2%)		70 (30.0%)	40 (17.2%)	
Low expression	25 (11.1%)	90 (40.0%)		31 (13.3%)	92 (39.5%)	

Data are shown as *n* (%). KPS, Karnofsky Performance Status.

**Figure 3:**
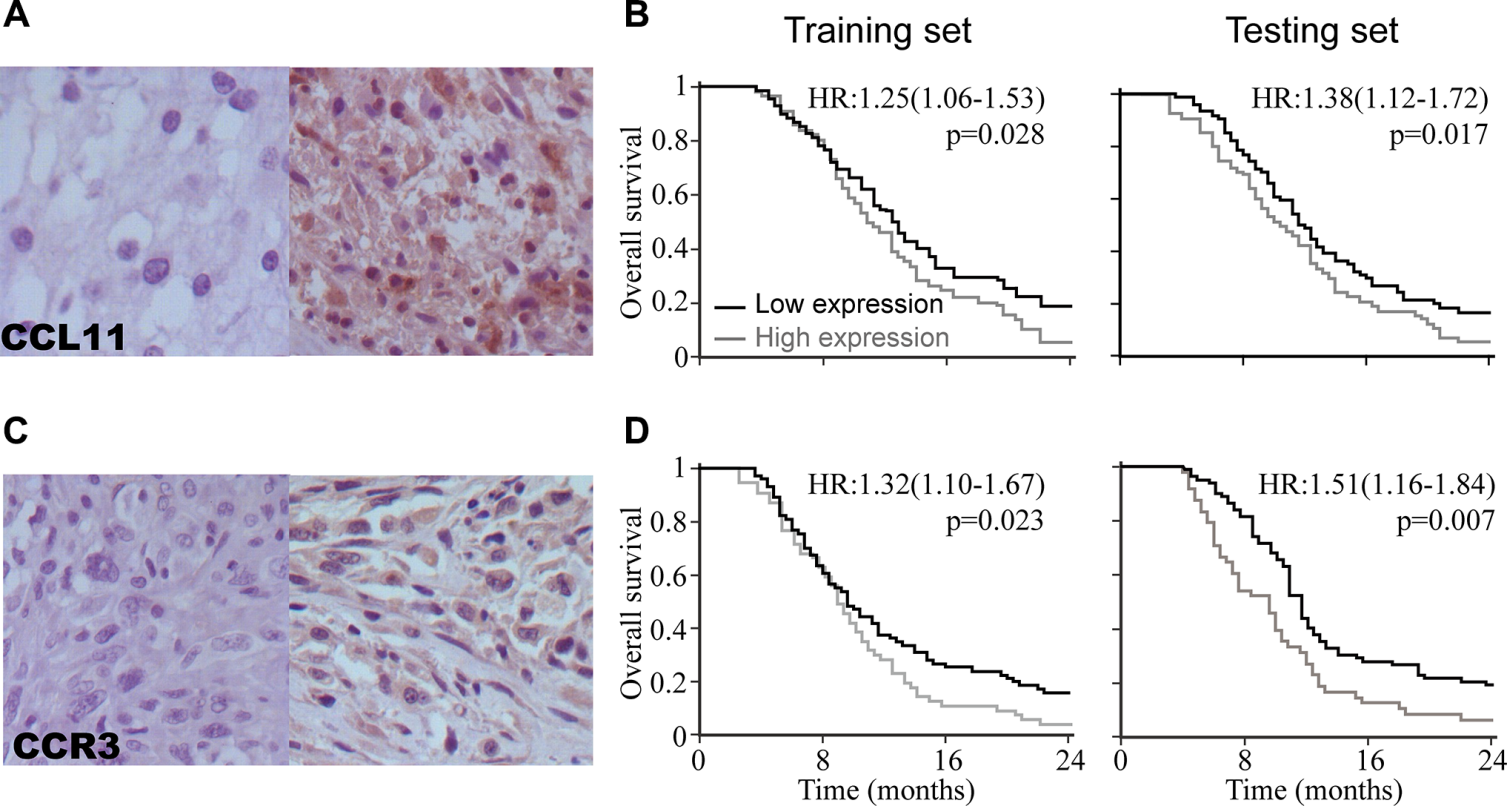
CCL11 and CCR3 were prognostic biomarkers for overall survival in GBM patients (**A**) Representative examples of CCL11 immunostaining. (**B**) Kaplan-Meier survival analysis for OS in training and test cohorts based on CCL11 expression. (**C**) Representative examples of CCR3 immunostaining. (**D**) Kaplan-Meier survival analysis for OS in training and test cohorts based on the CCR3 expression.

Immunoreactivity was observed in the tumor samples (Figure 3A, 3C). To assess the overall survival, ROC curve analysis was used to determine the cutoff scores for CCL11 or CCR3 in the training set [17]. The optimal cutoff values for CCL11 and CCR3 were 4.65 (*p* = 0.02) and 4.12 (*p* < 0.01), respectively. Accordingly, we selected a CCL11 expression score of 4 (> 4 vs. <= 4) as the cutoff value to categorize the GBM subjects into high- and low-expression subgroups in both the training and test sets. Similarly, an immunostaining score of 3 was the selected cutoff value for CCR3.

As shown in Table 1, there were no differences between patients in the CCL11 high-expression subgroup and low-expression subgroup in terms of age, gender, family history of cancer, previous low-grade tumor, extent of surgery, or diameter of tumor. Correlation analysis demonstrated that CCL11 was significantly associated with KPS scores and CCR3 expression in both the training and test set.

Kaplan-Meier analysis showed that high expression of CCL11 was strongly correlated with poor overall survival (OS) in the training set (HR, 1.25; 95% CI, 1.04–1.53; *p* = 0.03; Figure 3B) as well as the test set (HR, 1.38; 95% CI, 1.12–1.72; *p* = 0.02). In addition, high CCR3 expression was also a poor prognostic factor in both the training set (HR, 1.32; 95% CI, 1.10–1.67; *p* = 0.02; Figure 3D) and test set (HR, 1.71; 95% CI, 1.37–2.09; *p* = 0.01).

To avoid other confounds, the expression of CCL11 along with other clinic-pathological characteristics were examined in multivariate Cox analysis. As shown in Table 2, CCL11 and CCR3 were found to be independent prognostic biomarkers for OS. Age and KPS were also prognostic factors for OS. We failed to identify any other important variables as independent prognostic factors, including family history of cancer, tumor size, previous low-grade tumor, or extent of surgery.

**Table 2:** Multivariate Cox proportional-hazards analysis of overall survival in all GBM patients

Variables	Hazard ratio	95% CI	*p*
Age (> 60 vs. < = 60; years)	1.56	1.03–2.34	**0.034**
Gender (Male vs. female)	0.72	0.46–1.12	0.532
Family history of cancer (yes vs. no)	1.23	0.86–1.67	0.386
Previous low-grade tumor (yes vs. no)	1.36	0.75–2.57	0.471
KPS (< = 70 vs. > 70)	2.10	1.34–3.32	**0.002**
Extent of surgery (Complete resection vs. partial resection)	1.38	0.93–1.86	0.072
Diameter of tumor (> 5 cm vs. < = 5 cm)	1.44	0.98–2.09	0.056
CCL11 expression (high vs. low)	1.33	1.09–1.68	**0.023**
CCR3 expression (high vs. low)	1.46	1.12–1.97	**0.019**

### A prognostic model for GBM

**Figure 4:**
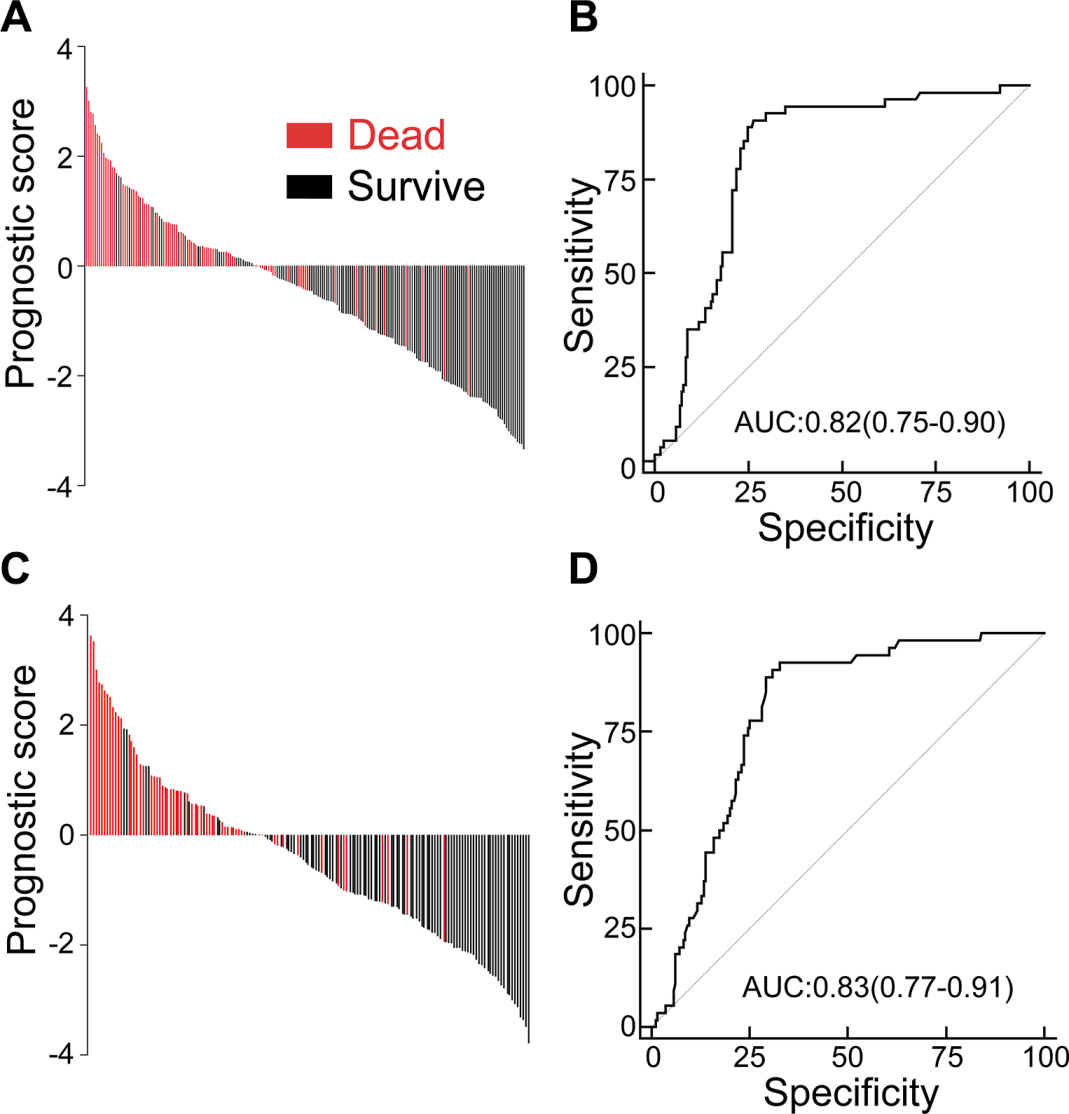
A prognostic model with CCL11 and CCR3 expression combined with KPS predicted one-year survival in GBM patients (**A**) Prognostic scores for all GBM patients in the training cohort. Each line represents one patient. (**B**)The receiver operating characteristics (ROC) curve and the corresponding values of area under curve (AUC) in the training set. (**C**) Prognostic scores for all patients in the test cohort. (**D**) ROC curve and the corresponding values AUC in the test set.

To predict the probability of 12-month survival in GBM patients, clinic-pathological characteristics including age, gender, family history of cancer, previous low-grade tumor, KPS, extent of surgery, diameter of tumor, and CCL11 and CCR3 immunostaining were included for leave-one-out cross-validation analysis. The combination of CCR3, CCL11 and KPS yielded the optimal sensitivity and specificity in the training set (Figure 4A). One discriminant formula was developed to assess the prognostic power taking account the strength of these parameters: prognostic score = (−0.452 × CCL11 histoscore) + (−0.395 × CCR3 histoscore) + ([KPS −68] × 0.018) + 3.794. According to this discriminant equation, patients in the training set were categorized into high-risk and low-risk subgroups with prognostic score = 0 as a cutoff value. Compared with low-risk patients, high-risk subjects had worse OS. The prognostic accuracy was further assessed by time-dependent ROC analysis (Figure 4B), and the sensitivity and specificity were 90.7% and 73.4%, respectively. The same cutoff values were also applied to the test cohort (Figure 4C). The ability of this prognostic classifier to discriminate survival patients from those with death within 12 months was significant (*p* < 0.001), with an area under the curve of 0.83 (95% CI, 0.77−0.91; Figure 4D).

## DISCUSSION

In this study, we discovered that CCL11 was upregulated in GBM using an antibody-based cytokine array system. Further analysis showed that the expression of both CCL11 and its receptor, CCR3, were increased in cell lines and clinical GBM samples. Moreover, our data demonstrated that both CCL11 and CCR3 promote the proliferation, migration, and invasion of cancer cells. In GBM patients, immunohistochemical analysis revealed that high expression of CCL11 and CCR3 were correlated with worse OS. In addition, we developed a prognostic formula combining CCL11 and CCR3 expression and KPS. The clinical application of this prognostic score could provide a valuable index of the probability of one-year survival for GBM patients.

GBM is a biologically complex disease with tumors composed of neoplastic glioma cells, endothelial cells, non-neoplastic neurons and glia, microglia, macrophages, neutrophils, and lymphocytes [1]. Secretion of CCL11 could come from any of these cell types and contribute to the development of GBM. To validate the elevation of CCL11 in clinical GBM tissues, it is necessary to clarify the source of CCL11. There are several studies suggesting that tumor cells themselves could be a possible source [8]. We found that cancer cells themselves could produce CCL11 and overexpress CCR3, indicating the activation of CCL11-CCR3 autocrine signaling in GBM. This result is consistent with a previous study, which showed that the *CCR3* gene was overexpressed in 60% of GBM [16]. Interestingly, the expression of CCR3 in lower-grade gliomas did not change significantly [16], suggesting different mechanisms were involved in the progression of GBM and lower-grade gliomas. This may explain why Moogooei et al. could not detect upregulation of CCL11 in glial tumors in a recently published paper [18]. In their study, only part of the samples came from GBM patients [18]. In addition, they chose to examine the levels of CCL11 in serum, while our data demonstrated it was the cancer cells that secreted CCL11. Thus, the levels of CCL11 might have been diluted in blood.

The chemokine network is a known target for treating GBM [19, 20]. Previous studies have shown that several chemokines and their receptors including CXCL12 [21], CXCL8 [22], CCL2 [23], RANTES [15], CX3CL1 [16], CCR4 [24], CXCR4 [25] and CX3CR1 [26] participate in tumorigenesis. In particular, CCR2 [27], CCR3 [16], and CCR5 [16, 28], three known CCL11 receptors, were all markedly upregulated in GBM. Our data indicates that CCL11 stimulates the proliferation, migration, and invasion of GBM cells. Furthermore, overexpression of CCL11/CCR3 was correlated with unfavorable OS. These effects are likely the result of multiple signaling pathways regulated by CCL11. Previous studies showed that the binding between CCL11 and CCR3 upregulated the expressions of VEGF, IL-8, PDGF-BB, and FGF [8–10]. In addition, it also induced phosphorylation of ERK1/2, MEK1 and STAT3 [5, 29]. Accordingly, CCL11 could act as: (1) an inflammatory chemokine contributing to the host response to neoplasia, (2) a proangiogenic factor promoting new vessel formation, (3) a general pro-inflammatory factor in response to tissue stress and/or necrosis, and/or (4) an autocrine growth factor released by cancer cells to promote proliferation, migration, and invasion [5, 8, 30].

The dysregulation of CCL11/CCR3 has been studied in several different diseases [7]. In fact, CCL11/CCR3 have been implicated as diagnostic biomarkers in gastric cancer [9], prostate cancer [11], and ovarian cancer [31], and as prognostic biomarkers in renal cell carcinoma [12], lymphoma [5] and ovarian cancer [32]. Here, we found CCL11/CCR3 were prognostic biomarkers for OS, which might also open new opportunities for exploring CCL11/CCR3 in GBM therapy. In fact, a number of approaches including monoclonal antibodies and small molecule receptor antagonists/inhibitors targeting CCL11 or CCR3 have been tested and some of them have progressed into the clinic [8, 33]. Bertilimumab, a humanized monoclonal antibody against CCL11, is currently in clinical trials for treating severe allergic disorder, vernal keratoconjunctivitis, and inflammatory bowel disease [34]. BMS-639623 and GSK766994, two potent CCR3 antagonists, are also in clinical trials for treating asthma [33]. Accordingly, it is conceivable that a clinical trial targeting against CCL11 and/or CCR3 in GBM can be conducted if our study can be validated by other groups.

Combating GBM still remains a major clinical challenge, and, thus far, results have been rather disappointing. Molecular biomarkers would help to identify those patients at high risk of death. When examining the ability to predict one-year survival using logistic regression analysis, the expression of CCR3 and CCL11 along with KPS were found to be accurate predictors. Time-dependent ROC analysis revealed that the accuracy rate of one-year survival prediction was 82% in the training cohort. A test cohort of 233 patients with an accuracy rate of 83% further verified this result. These data suggest that a clinical application of CCL11 and CCR3 expression combined with KPS might provide a valuable index of the probability of one-year survival. Successful predictions using these biomarkers may help doctors design more aggressive treatments to extend the survival of GBM patients.

In summary, the chemokine, CCL11, along with its cognate receptor, CCR3, have been identified as major factors influencing GBM tumor development. Furthermore, a prognostic index, using CCL11 and CCR3 expression combined with KPS is remarkably valuable in predicting one-year survival in GBM patients. These findings may not only have important implications for the development of anti-cancer therapies, but also provide powerful biomarkers for prognosis in GBM, which, to date, have only had limited success.

## MATERIALS AND METHODS

### Cell lines and cell culture

The human glioblastoma cell lines U251MG, A172 and U87MG were obtained from the Institute of Biochemistry and Cell Biology, Chinese Academy of Sciences, Shanghai, China within six months of being used. All cell lines were routinely maintained at a humidified atmosphere containing 5% CO_2_ at 37°C, in DMEM (Gibco, Carlsbad, CA) containing 10% fetal bovine serum (FBS, Invitrogen, USA), 1% antibiotics (penicillin and streptomycin) and 1% HEPES buffer solution. Normal human astrocytes (NHA) were obtained from the Sciencell Research Laboratories (Sciencell, USA) and cultured under conditions as instructed by the manufacturer.

### Patients and clinical samples

In this study, a total of 458 pathologically proven primary GBM patients (255 male, 203 female) were enrolled from the First Hospital of Harbin Medical University (Harbin, China) and the General Hospital of Beijing Military Command (Beijing, China) between January 2000 and December 2014. Patients without tumor samples or preoperative death were excluded. All samples were immediately acquired after surgery. Of the overall cohort, 225 patients were randomly assigned by computer to the training set, and the remaining 233 patients were assigned to the test set. The observation time in these patients was defined as the interval between the initial diagnosis and the last time of contact (either last follow up or death). Overall survival (OS) was defined as the period from the date of initial diagnosis to death of the same subject, and was used for analyses. We state that the authors followed the principles outlined in the Declaration of Helsinki for human or animal experimental investigations and obtained appropriate institutional review board approvals from both hospitals.

### Cytokine antibody array

Total protein was extracted from clinical samples using tissue protein extraction reagents (Anji Biotech, China). An antibody-based cytokine array system was used to detect the levels of growth factors and cytokines in GBM tissue and adjacent noncancerous tissue. The experiment was carried out with a RayBio Human Cytokine Array kit (Cat. Number: AAH-CYT-G6, RayBio, USA) to detect the expression of 60 cytokines according to the manufacturer's recommendations. Signal intensity was quantified by light densitometry.

### RNA isolation and qRT-PCR

Total RNA was extracted from cell lines and tumor specimens using Trizol reagent (Invitrogen, USA) according to the manufacturer's instructions. *CCL11* and *CCR3* mRNA expression was measured by qRT-PCR using an ABI7900HT instrument (Life Tech, USA). *GAPDH* was used as an internal control. The primers were as follows: *CCL11*: forward primer 5′-ACACCTTCAGCCTCCAACAT-3′ and reverse primer 5′- GGTCTTGAAGATCACAGCTT-3′; *CCR3*: forward primer 5′- TCGTTCTCCCTCTGCTCGTT-3′ and reverse primer 5′-GCCGGATGGCCTTGTACTTT-3′; and *GAPDH*: forward primer 5′-GGTATGACAACGAATTTGGC-3′ and reverse primer 5′- GAGCACAGGGTACTTTATTG -3′. Relative expression was examined by the standard curve method. All standard curves were linear in the required range with acceptable correlation coefficients. Specific gene mRNA levels were given as ratios to *GAPDH* mRNA levels. All experiments were done in triplicate.

### Anti-CCL11 treatment and *CCR3* gene silencing

For anti-CCL11 treatment, cancer cells were incubated with neutralizing human CCL11 monoclonal antibody or a mouse monoclonal IgG1 isotype control (R&D system, USA). Tumor cells were harvested at various time-points for analysis, each experiment was repeated five times.

For *CCR3* gene silencing, custom-made plasmids carrying *CCR3*-shRNA were obtained (Anji Biotech, China). For transfection, cancer cells were cultured overnight at the logarithmic growth phase, then transfected with the studied vectors or negative control with Lipofectamine 2000 (Invitrogen, USA) according to the manufacturer's instructions for 72 hours. Stable transfected clones were validated by qRT-PCR and immunoblotting.

### Immunohistochemistry

Immunohistochemical staining was performed by the avidin-biotin-peroxidase complex method. Briefly, 4 μm serial sections were cut from formalin-fixed paraffin-embedded tumor tissues. After rehydration and microwave antigen retrieval, monoclonal antibodies against human CCL11 (R&D Systems, USA) and CCR3 (R & D Systems, USA) were applied to slides, incubated at 4°C overnight, and followed with secondary antibody incubation at 37°C for 30 min. Staining was carried out with DAB and counter-staining with Mayer's hematoxylin. Negative control slides with the primary antibodies omitted were included in all assays.

The staining was evaluated based on previously reported guidelines [35, 36]. Staining intensity was scored as follows: no staining at all (score 0), faint staining (score 1), moderate staining (score 2) and strong staining (score 3). The distribution of the protein studied was defined as the percentage accounting for the whole area in the section: 0% (score 0), 1–25% (score 1), 26–50% (score 2), 51–75% (score 3) and 76–100% (score 4). Total scores were calculated by combining the evaluation of staining intensity and staining distribution. The results of staining were independently evaluated by two researchers (M.T. and L.C.). If both of them agreed with the result, the score was determined. If discrepancies appeared, a third researcher (Y.J.) would participate in the evaluation and work together to get a final score.

### Cell proliferation assays

Cells (3000 cells/well) were dispensed in 100-μL aliquots into a 96-well plate. 20 ml of 3-(4,5-dimethylthiazol-2-yl)-5-(3-carboxymethoxyphenyl)-2-(4-sulfophenyl)-2H-tetrazolium inner salt (MTS, Promega, USA) was added into each well after cells were cultured for 1, 2, 3, 4, 5 and 6 days. The cells were then incubated at 37°C for 4 h in a 5% CO_2_ incubator. Optical density for the cell viability was obtained at a wavelength of 490 nm using spectrophotometric analysis. All experiments were conducted three times.

### Wound healing assay

Briefly, cells were grown to 80% confluence in 6-well plates (Corning, USA). An artificial wound was scratched using a standard 200 μL pipette tip, after which the cells were further incubated. Migration into the scratched area was documented. At varying hours, cells were photographed using an inverted microscope, and the widths of the wound lines were measured. Scratch closure was evaluated relative to the total area of the wound. Each experiment was performed in triplicate.

### Invasion assay

Cell invasion assays were performed using 24-well transwell (8-μm pore size; Minipore, USA) coated with Matrigel (BD Biosciences, USA). In total, 1 × 10^5^ cells were suspended in 100 μL Dulbecco's modified Eagle medium with 1% fetal bovine serum and added to the upper chamber, and 600 μL Dulbecco's modified Eagle medium with 10% fetal bovine serum was placed in the lower chamber. After 48 hours of incubation, the Matrigel and the cells remaining in the upper chamber were removed by cotton swabs. Cells on the lower surface of the membrane were fixed in 4% paraformaldehyde and stained with Giemsa. Cells in five microscopic fields (at 200× magnification) were counted and photographed. All experiments were performed in triplicate.

### Statistical analysis

The association between clinicopathological features and different biomarkers were analyzed using χ2 test, Student's *t* test, or Fisher's exact test as appropriate. Cox proportional hazards regression models were used to assess prognostic values of protein expression. Kaplan-Meier analysis and log-rank test were performed to evaluate the overall survival, hazard ratios (HR) and 95% confidence interval (CI). Data are expressed as mean ± s.e.m. Quantitative data between different groups were compared with one-way analysis of variance (ANOVA), followed by LSD method for post-hoc test comparisons.

To determine the cutoff values of CCL11 and CCR3 for OS, receiver operating characteristic (ROC) curve analysis was performed in the training cohort as previously reported [17]. Briefly, by maximizing the combination of specificity and sensitivity; minimizing the overall error and the distance of the top-left corner of ROC curve to the cutoff value, the optimum cutoff point was selected. Here, the clinical outcomes were classified into two categories according to survival conditions; i.e. death because of GBM verses all the other clinical outcomes (such as survival, censored, or death but from other causes).

To estimate the variables of immunoreactive markers or clinicopathological features that may contribute to the prognostic significance, those significant differences between patients that were dead and alive 12 months after initial diagnosis were evaluated by logistic regression analysis. The significant factors obtained were used for the training set to construct classifiers. The classifiers were examined using leave-one-out cross-validation within cases of the training cohort [37]. A discriminant model for the prognosis was derived according to each type combination of these factors. The equation of the optimal combination was used to predict the probability of 12-month survivals in the test cohort. In this study, individual patients' prognostic factors were multiplied by the coefficients as follows: prognostic score = (−0.452 × CCL11 histoscore) + (−0.395×CCR3 histoscore) + ([KPS −68] × 0.018) + 3.794, where KPS-68 means Karnofsky performance score at diagnosis minus 68. ROC curves were generated to compare the predictive sensitivity, specificity, and area under the curve. Statistical significance was set at *P* < 0.05 (two-tailed).
